# Enhanced radiation shielding efficiency of polystyrene nanocomposites with tailored lead oxide nanoparticles

**DOI:** 10.1038/s41598-024-69510-4

**Published:** 2024-08-28

**Authors:** Mona M. Gouda, Ahmad Firas Osman, Ramadan Awad, Mohamed S. Badawi

**Affiliations:** 1 Physics Department, Faculty of Science, Alexandria University, Alexandria, Egypt; 2 Lebanese Atomic Energy Commission, National Council for Scientific Research, Beirut, Lebanon; 3 Department of Physics, Faculty of Science, Beirut Arab University, Beirut, Lebanon; 4 Department of Basic Sciences, Faculty of Computer Science and Artificial Intelligence, Pharos University in Alexandria, Alexandria, Egypt; 5 Faculty of Science, Alamein International University, Alamein City, Matrouh Governorate Egypt

**Keywords:** PbO nanoparticles, Polystyrene nanocomposites, Gamma-ray shield, Nanoscience and technology, Physics

## Abstract

In this study, we investigated a novel polymer nano-composite, PS-PbO, containing two distinct nano-sizes of lead oxide nanoparticles (PbO-A and PbO-B), in addition to the bulk size (PbO-K). These nanoparticles were embedded separately in a polystyrene (PS) matrix at different weight percentages (10%, 15%, 25%, and 35%) using roll mill mixing and compressing molding. Our evaluation focused on the radiation attenuation ability of PS-PbO and the effect of particle size, considering gamma-ray energies ranging from 0.06 to 1.3 MeV (from sources like ^241^Am, ^133^Ba, ^137^Cs, and ^60^Co). The linear attenuation coefficient (LAC) was determined by analyzing samples of the synthesized composite with different thicknesses. Then, various shielding parameters were calculated, including total molecular, atomic, and electronic cross-sections (*σ*_*mo*l_, *σ*_*atm*_, *σ*_*e*l_), as well as the effective atomic number and the electron density (*Z*_*eff*_ and *N*_*eff*_). Surprisingly, modifying PbO particle sizes had a significant impact on shielding efficiency. For instance, the composite with 25 wt% of the smallest PbO-B particles showed a 26.7% increase in LAC at 0.059 keV compared to the composite with 25 wt% of PbO-K (larger particles). Notably, the LAC peaked at low energy (0.059 keV), close to the K-edge of Pb, where interaction is directly proportional to Z^4^. With increasing PbO concentrations, the LAC of PS-PbO composites increased steadily. Additionally, as PbO concentration increased, the composite’s effective atomic number *Z*_*eff*_ and the electron density *N*_*eff*_ increased, leading to a greater total Gamma-ray interaction cross-section. Furthermore, when comparing the Half-Value Layers of the novel nanocomposite to traditional lead shielding, a 70% reduction in mass was observed. Notably, the composite containing the smallest nano-size of PbO exhibited the highest radiation-shielding efficiency among all combinations and could therefore be used to create inexpensive and lightweight shields.

## Introduction

The need for effective radiation shielding is becoming increasingly important in various fields, such as medical and aerospace industries, nuclear power plants, and space exploration. Traditional radiation shielding materials, such as lead and concrete, have limitations in terms of weight, cost, and environmental impact. Given their unique characteristics, such as high mechanical strength, low density, and tunable radiation absorption, the emerging field of polymer nanocomposites offers a promising alternative to radiation shields^1,2^. In recent years, significant advancements have been made in the development of polymer nanocomposites for radiation shielding^3,4^. To overcome the weight and toxicity of the heavy metal, efforts were made to replace lead (Pb) with lead oxide (PbO)^5^, which, when added to polymers, improves the radiation shielding capability of such composites^6–9^. Polystyrene (PS) is a thermoplastic polymer with high levels of rigidity, transparency, and viscosity. PS is used for a variety of applications, including electrical and thermal insulation, as a result of its ease of production, low cost, and resistance to alcohols, alkalis, oils, and acids^10^.

Turhan et al.^11^ prepared a polymer composite by mixing unsaturated polyester resin, Methyl Ethyl Ketone Peroxide MEKP, and Cobalt Octoate. After this process, hematite was added to the polymer matrix as a phase material at different ratios (25%, 50%, 75% and 100%). Gamma-ray radiation attenuation parameters were studied for the energy range from 59.5 to 1408.0 keV. The results showed that the mass attenuation coefficient of hematite doped composites with a percentage between 25 and 100%, for the energy range of 59.5–1408.0 keV, was changed between 0.3301–0.0551, and 0.5461–0.0537 cm^2^ g^−1^. The hematite (100%) polymer composite is reportedly a more effective shield than other produced composites.

El-Khatib et al.^12^ prepared Dimethylpolysiloxane (Silicone Rubber SR) incorporated with micro and nano sizes lead oxide at various weight percentages to study the effect of particle size and concentration of PbO particles on radiation attenuation parameters. The matrix was tested against ^241^Am, ^137^Cs, ^60^Co, ^133^Ba, and ^152^Eu gamma sources. The influence on the attenuation capacity of SR-PbO demonstrates that the addition of PbO improves the linear attenuation coefficient significantly, particularly at low gamma energies. Furthermore, The nano PbO composites presented 22–30% higher linear attenuation coefficients compared to micro PbO composites.

PS-b-polyethylene glycol and PS-b-polyethylene glycol-boron nitride doped with PbO were investigated by Cinan et al.^13^ as nanocomposite shields. The composite was exposed to a ^152^Eu source and radiation attenuation parameters were evaluated for photon energy between 121.78 and 1408.01 keV. The obtained results indicated that the linear attenuation coefficient *μ* decreases with increasing photon energy. Moreover, as the PbO percentages in the composites increased, their radiation protection capacities were improved.

Kiani et al.^14^ investigated the gamma-ray shielding ability of Epoxy-Clay-PbO nanocomposites ECPNC prepared by molding techniques. They evaluated the attenuation coefficients using the photon energy range of three gamma sources ^192^Ir, ^137^Cs and ^60^Co. The results showed that the linear attenuation coefficient of ECPNC increased with increasing PbO weight percent and decreased with increasing gamma energy. Additionally, ECPNCs containing 30 wt% PbO performed 47% better than concrete.

Mahmoud et al.^15^ studied the influence of particle size on the radiation attenuation capabilities of composite. The combustion method was used to prepare lead oxide nanoparticles. PbO-K and PbO with mass fractions of 10 and 50% were incorporated into high-density polyethylene (HDPE). The prepared composite was evaluated for radiation shielding efficiency using four standard radioactive point sources (^241^Am, ^133^Ba, ^137^Cs, and ^60^Co). The results indicated that, as the weight percentage of PbO in the HDPE matrix increased, the linear and mass attenuation coefficients increased significantly for all energies. Additionally, at the same weight fraction, an increase in the attenuation coefficient values was observed for PbO NPs composites compared to PbO-K composites.

Mostafa et al.^16^ prepared PbO–Sb_2_O_3_–B_2_O_3_–CuO glass using a conventional melt technique and examined the system as a gamma and neutron shield. The mass attenuation coefficient values of PS PSBC1, PSBC2, PSBC3, PSBC4, and PSBC5 glasses at 0.015 MeV were 31.2, 35.6, 44.9, 55.1, and 66.3 cm^2^/g, respectively. Indicating that the PSBC5 sample with a higher density (4.81 g/cm^3^) provided the superior gamma shield. Another glass system (30Tl_2_O_3_ + 10Li_2_O + (60 − x)B_2_O_3_ + xSm_2_O_3_) where x represents the percentage of boron trioxide with samarium (III) oxide (x = 0, 0.2, 0.4 and 0.6 wt%), was examined by Saudi et al.^17^ using a narrow beam geometry and experimental gamma-ray transmission method at 0.081, 0.356, 0.662, 1.173 and 1.33 MeV. The results of the mass attenuation coefficient were respectively 0.639, 0.647, 0.655, and 0.663 cm^2^/g at 0.081 MeV. The most effective shielding properties were found for the BTLSm4 sample with x = 0.6 t%. Zakaly et al.^18^ studied glass fabrication using ceramic and porcelain recycled waste and lithium niobate as nuclear radiation shields. They investigated the glass form of xLiNbO_3_–(40 − x) Waste with different LiNbO_3_ contents of x = 8, 16, 20, 24, 32 wt% for photon energies in the range of 81–2614 keV. The results showed that as the LiNbO_3_ concentration increases from 8 to 32% (wt.), the material density increases from 5.570 to 5.920 g/cm^3^. The Waste 32 sample with 32 wt% LiNbO_3_ has the greatest LAC values proving that it is the best shield out of the fabricated Waste-x samples.

This research developed a new gamma-ray shielding composite material that is economical, cost-effective, and simple to process. This material can be employed in a variety of applications, especially wall shields in radiology, radiotherapy, and nuclear medicine departments. Using a variety of radioactive sources, the gamma-ray shielding capabilities of PS-PbO composites over the energy range of 0.06–1.3 MeV were evaluated. In addition, the particle size effect of PbO as a filler was investigated by adding various amounts (10, 15, 25, and 35 wt%) of either bulk or nanoparticles PbO (78 and 52 nm) in the PS polymer matrix. This research aims to determine the optimal size of PbO particles that can be incorporated into the PS polymer to produce lightweight composites with exceptional radiation shielding performance. This article provides key challenges and opportunities in polymer nanocomposites for radiation shielding and reviews the current state-of-the-art in this rapidly growing field.

## Material and methods

### Materials

Polystyrene (PS) was provided by the Egyptian Styrene & Polystyrene Production (ESPP) Company E-STYRENICS (Alexandria, Egypt) and used as a matrix for the composite. PS density is 1.03 g/cm^3^ and the melt flow rate is 11.1 g/10 min. Lead (II) oxide (PbO) with a purity of 99.0% was acquired from Sigma-Aldrich and used as a filler after being converted to PbO nanoparticles. PbO density is 9.53 g/cm^3^ and the molecular mass is 223.20 g/mole.

### PbO nanoparticles synthesis

The synthesis of nanoparticles of lead oxide was performed using Retsch, PM 100 (planetary ball miller at high speed). The milling was processed in a 250-ml zirconium oxide container using zirconium balls. The ball-to-powder weight ratio was 10:1. The milling procedure was executed at four distinct times (15, 30, 60, and 120 min), and the rotational speed was fixed at 400 rpm. Every milling cycle comprises a grinding period of five minutes followed by a rest of one minute to avoid overheating and particle agglomeration. PbO particles with nanoscale dimensions of 78 and 54 nm, respectively, have been termed PbO-A and PbO–B after being milled for 30 and 60 min.

### PS-PbO composite preparation

Using compression molding, the PS-PbO composite was fabricated by incorporating separately different weight fractions (0, 10, 15, 25, and 35 wt%) of both PbO nanoparticles (A), (B) and the bulk PbO. Polystyrene and PbO particles were proportionately weighed and thoroughly mixed at 200 °C in XK400, Shandong (a two-roll mill mixer). The mixing time was set to 30 min and the roll velocity was set to 50 rpm to achieve acceptable additive dispersion and composite homogenization. The resulting paste was then sandwiched among both Teflon layers in a stainless steel mold (25 × 25 × 0.3 cm^3^) to create a smooth surface. The sample was then hydraulically pressed at 200 °C and 20 MPa for 15 min. Following sintering, the composite sample was progressively cooled to room temperature under compression for 15 min. For the evaluation of the radiation properties of the composite, five discs with 8.5 cm diameter were snipped from the sample and used for the required tests. As shown in Table 1, using the described method, thirteen specimens were collected and coded.

**Table 1:** Composition and sample codes of examined materials.

Sample code	Description
PS	Pure polystyrene
PbO-K	Yellow powder of PbO
PbO-A	78 nm nanoparticle of PbO
PbO–B	52 nm nanoparticle of PbO
PS-PbO-K 10	10 wt% of PbO-K embedded in PS
PS-PbO-K 15	15 wt% of PbO-K embedded in PS
PS-PbO-K 25	25 wt% of PbO-K embedded in PS
PS-PbO-K 35	35 wt% of PbO-K embedded in PS
PS-PbO-A 10	10 wt% of PbO-A embedded in PS
PS-PbO-A 15	15 wt% of PbO-A embedded in PS
PS-PbO-A 25	25 wt% of PbO-A embedded in PS
PS-PbO-A 35	35 wt% of PbO-A embedded in PS
PS-PbO-B 10	10 wt% of PbO-B embedded in PS
PS-PbO-B 15	15 wt% of PbO-B embedded in PS
PS-PbO-B 25	25 wt% of PbO-B embedded in PS
PS-PbO-B 35	35 wt% of PbO-B embedded in PS

### Sample density measurement

Based on Eq. (1), the density of the sample was calculated using the Archimedes principle.

1 $$\rho = \left[ {\frac{M}{{M_{a} - M_{w} }}} \right]\rho_{w} ,$$

where *ρ*_*w*_ is the density of water (1.00 g/cm^3^). *M*, *M*_*a*_, and *M*_*w*_ are the masses of the sample when it was on the balance, swinging on the arm in the air, and laying on the arm in the water, respectively.

For mass evaluation, an M-120 balance (Denver) having a sensitivity of 10% of the mg was utilized. Equation (2) was used to calculate the composites’ density theoretically^19^.

2 $$\rho_{T} = \left[ {\frac{100}{{\frac{{M_{m} }}{{\rho_{m} }} + \frac{{M_{f} }}{{\rho_{f} }}}}} \right],$$

where *M*_*m*_ is the PS weight percent, *M*_*f*_ is the PbO weight percent, *ρ*_*m*_ is the PS volumic mass, and *ρ*_*f*_ is the PbO volumic mass.

### Measurements of gamma-ray attenuation coefficients

**Figure 1:**
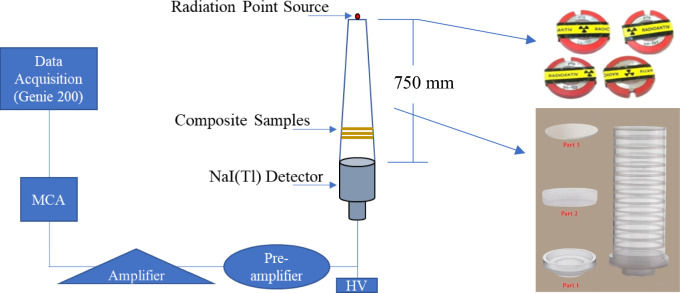
Schematic presentation of the gamma-ray attenuation setup.

Figure 1 represents the gamma-ray attenuation setup using a narrow beam^20^ and NaI(Tl) scintillation detector^21–23^. The measurements were performed in the Radiation Physics laboratory, Faculty of Science, Alexandria University, Alexandria, Egypt. Four radioactive sources of ^241^Am, ^133^Ba, ^137^Cs, and ^60^Co from PTB were used for radiation measurements. The sources are classified according to Gamma energy as shown in Table 2. The sources were positioned 75 cm axially away from the detector. The spectrometry system consists of an HV power that supplies the detector with the required voltage to function. A preamplifier that reduces noise sources, shapes and amplifies the pulse. A signal amplifier that multiplies the signal by 1000 or more. Multichannel analyzer (MCA) for processing and analysis. Genie 2000 which is a Canberra software of ISO 9001 was used for data acquisition and analysis^24–26^.

**Table 2:** The radioactive sources and their related gamma energies.

Radioactive source	Photon energy (MeV)
^241^Am	0.05953
^133^Ba	0.35601
^137^Cs	0.66166
^60^Co	1.17323
	1.33250

The LAC (cm^−1^), illustrates the portion of incident photons that are attenuated per unit thickness of a material in a monoenergetic beam. It accounts for all pertinent interactions, including the photoelectric effect, Compton scattering, and pair production. The intensity or count rate at a thickness *x* (cm) is computed using the Beer–Lambert law Eq. (3).

3 $$N = N_{0} e^{ - \mu x} ,$$

where *µ* is the LAC,* N*_*0*_ is the primary count rate, and *N* is the count rate at depth *x* (cm).

Equation (4) describes the calculation of *μ* (cm^−1^) for each composite.

4 $$\mu = \frac{1}{x}{\text{Ln}}\left( \frac{N_{0}}{N} \right),$$

At the beginning of each experiment, the initial count rate *N*_*0*_ was determined by recording counts without any attenuator between the beam and the detector. Afterward, five disk specimens per sample, with around 4 mm thickness each, were successively positioned between the beam and the detector, and the count rates were registered. *μ* was calculated from the slope of Ln(*N*_*0*_/*N*_*x*_) versus *x* of the charted outcomes.

NIST standard reference databases were used to estimate the theoretical values of the composite linear attenuation coefficient. Partial *µ*_*i*_ for the single constituent of the composite were extracted from XCOM. Taking into account every elemen’s influence to the total photon interaction, the cumulative linear attenuation coefficient was then calculated using Eq. (5):

5 $$\mu = \sum\limits_{i} {w_{i} \left( {\mu_{i} } \right)} ,$$

where *µ*_*i*_ is the partial mass attenuation coefficient of a constituent in the composite and *w*_*i*_ represents its weight fraction. The weight fraction *w*_*i*_ is defined as:^27^

6 $$w_{i} = \frac{{n_{i} A_{i} }}{{\sum\nolimits_{i} {n_{i} A_{i} } }},$$

where *n*_*i*_ is the number of atoms of the ith constituent and *A*_*i*_ is its atomic weight.

Essential variables characterizing the interaction likelihood of photons with matter are cross-sections. Equation (7) gives the total molecular cross-section *σ*_*mol*_ (cm^2^/molecule):^28^

7 $$\sigma_{mol} = \frac{{\left( {\mu /\rho } \right)}}{{N_{A} }}\sum\limits_{i} {n_{i} A_{i} } ,$$

Since Eq. (8) corresponds to the total atomic cross-section *σ*_*atm*_ (cm^2^/atom),

8 $$\sigma_{atm} { = }\frac{{\left( {\mu /\rho } \right)_{mix} }}{{N_{A} \sum\nolimits_{i} {\left( {\frac{{w_{i} }}{{A_{i} }}} \right)} }} = \frac{{\sigma_{mol} }}{{\sum\nolimits_{i} {n_{i} } }},$$

where *N*_*A*_ is the number of Avogadro (6.024 86 × 10^23^ mol^−1^).

In addition, Eq. (9) gives the total electronic cross-section *σ*_*e*_ (cm^2^/electron):^29^

9 $$\sigma_{el} { = }\frac{1}{{N_{A} }}\sum\limits_{i} {\frac{{f_{i} A_{i} }}{{Z_{i} }}} \left( {\mu /\rho } \right)_{i} ,$$

where *Z*_*i*_ represents the atomic number of the ith component, and *f*_*i*_ is its proportional abundance.

According to Eq. (10), the effective atomic number *Z*_*eff*_ can be calculated knowing *σ*_*atm*_ and *σ*_*el*_:^30^

10 $$Z_{eff} = \frac{{\sigma_{atm} }}{{\sigma_{el} }},$$

Finally, the number of electrons per unit mass is represented by the effective electron density *N*_*eff*_ measured in electrons/g × 10^22^, and calculated from Eq. (11):^31^

11 $$N_{eff} { = }\tfrac{{N_{A} }}{{\sum\nolimits_{i} {n_{i} A_{i} } }}Z_{eff} \sum\limits_{i} {n_{i} } .$$

## Results and discussion

### Samples densities

Sample densities were obtained from Eq. (1). Due to the high density of PbO, the results shown in Table 3 indicate that the composites’ density got higher with increasing PbO content, as predicted. Also decreasing PbO nano-size increases the composite density. At high concentrations of PbO (25 and 35 wt%), reducing particle size helps to fill the pores caused by insufficient PS to completely cover the PbO powder's surfaces, thereby increasing composite density^32^.

**Table 3:** Sample densities.

Sample	Density (g/cm^3^)
PS	1.032 ± 0.001
PS-PbO-K 10	1.080 ± 0.046
PS-PbO-K 15	1.170 ± 0.009
PS-PbO-K 25	1.250 ± 0.011
PS-PbO-K 35	1.401 ± 0.026
PS-PbO-A 10	1.123 ± 0.010
PS-PbO-A 15	1.199 ± 0.038
PS-PbO-A 25	1.279 ± 0.035
PS-PbO-A 35	1.425 ± 0.017
PS-PbO-B 10	1.134 ± 0.001
PS-PbO-B 15	1.200 ± 0.043
PS-PbO-B 25	1.347 ± 0.063
PS-PbO-B 35	1.469 ± 0.083

## Summary of previous nano-composite characterization results

In previous research conducted by Osman et al.^33,34^, the characterization of PbO particles milled for various amounts of time as well as the properties of polystyrene PbO nanocomposites was discussed in depth.

Two PbO phases, the yellow massicot phase β-PbO and the red litharge phase α-PbO, were revealed by XRD analysis of PbO milled particles. The ratio of PbO phases decreases as milling time increases, indicating a partial phase transition caused by milling pressure on the particles. TEM micrographs of the PbO particles demonstrate that the bulk PbO has an orthorhombic structure and is shaped differently than the milled PbO particle, which has a tetragonal structure, confirming the XRD result.

Using XRD and TEM, the crystallite size of milled PbO particles was determined, and a high degree of congruence was observed between the two methods of measurement.

**Figure 2:**
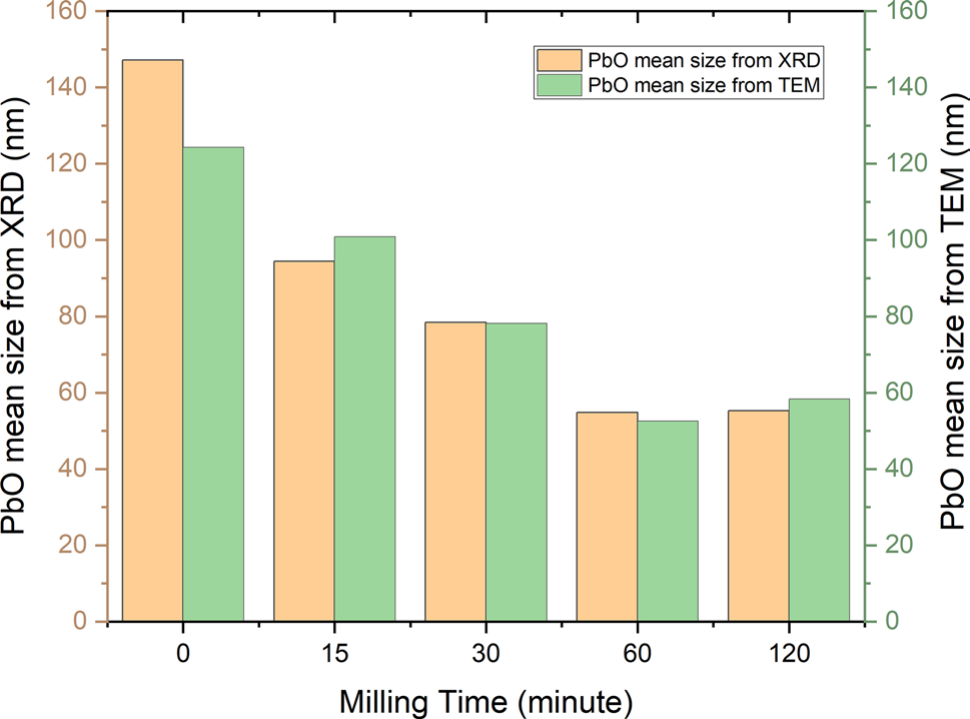
Results of PbO particle size calculated by XRD and TEM as a function of the milling time.

Figure 2 displayed the particle size for each milling time, the dimensions of the PbO particles decreased with increasing grinding time, reaching around 52 nm after 60 min, followed by a slight increase at 120 min.

Dynamic Light Scattering (DLS) evaluation turned into applied to research similarly the dimensions changes, distributions, and aggregations that took place all through the mechanical milling process. According to the results, PbO particles milled for 30 and 60 min exhibited a high intensity distribution for the lowest size detected, thereby exhibiting low aggregation. These two PbO types nanoparticle, designated PbO-A and PbO-B, were selected for this study.

Milling settings influenced the resulting nano-sizes of PbO. Varying the milling duration while maintaining a constant rotational speed and cooling break time in dry mode resulted in varied nano-sizes. The ball milling of PbO powder was performed several times under the same conditions to produce the required quantity of each PbO nano-size for the composite. Notably, the ball milling method demonstrated excellent precision, stability, and convergence, as evidenced by the results.

**Figure 3:**
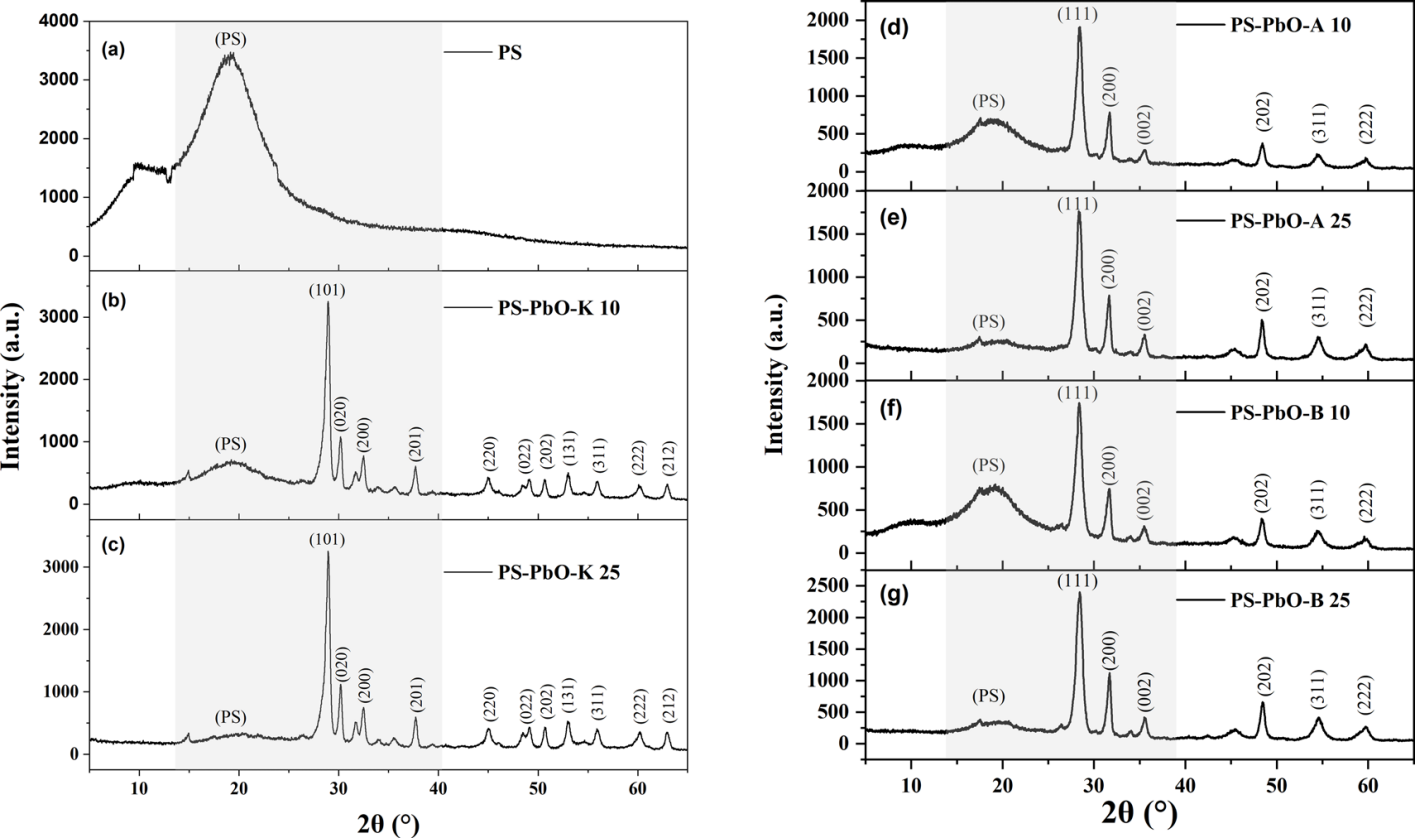
XRD patterns of (**a**) PS, (**b**) PS-PbO-K 10, (**c**) PS-PbO-K 25, (**d**) PS-PbO-A 10, (**e**) PS-PbO-A 25, (**f**) PS-PbO-B 10, and (**g**) PS-PbO-B 25.

Regarding the nanocomposites PS-PbO-A and PS-PbO-B, The XRD results shown in Fig. 3, revealed the presence of the tetragonal phase peaks of α-PbO in addition to the characteristic peaks of PS. The incorporation of PbO additives increased the ratio of α-PbO to PS. As a result, the intensity of PbO peaks increased while the intensity of PS characteristic peaks declined, resulting in a reduction of the amorphous nature and enhancement of the crystalline nature of the nanocomposite. In the produced composites, neither the peaks of PbO nor the characteristic peaks of PS were altered, indicating the absence of any chemical interaction and reflecting the adsorption of the metal oxide within the polymer matrix.

### Linear attenuation coefficient

The LAC *μ* (cm^−1^) was employed to characterize the radiation attenuation behavior of the composite. High atomic number and high physical density of absorbing materials generally increase the LAC, while it decreases with increasing incident beam energy, except at the K-edge. Table 4 shows the measured LAC values for PS, PS-PbO-K, PS-PbO-A, and PS-PbO-B for the γ-ray energies used in this study. The experimental values of *μ* for PS-PbO-K are compared to theoretical values obtained from XCOM and the discrepancy (Δ%) is displayed in Table 4. Also, the relative increase (δ%) due to the particle size of PbO is calculated and listed.

**Table 4:** Experimental and theoretical results of the LAC (*μ)* of PS, PS-PbO-K, PS-PbO-A, and PS-PbO-B.

Energy (MeV)	0.059	0.356	0.661	1.173	1.332
XCOM Theoretical *µ* (cm^−1^)					
PS	0.1839	0.1107	0.0856	0.0653	0.0611
PS-PbO-K 10	0.6384	0.1317	0.0916	0.0679	0.0635
PS-PbO-K 15	0.9331	0.1512	0.1004	0.0733	0.0685
PS-PbO-K 25	1.5125	0.1799	0.1096	0.0779	0.0726
PS-PbO-K 35	2.2743	0.2221	0.1254	0.0868	0.0807
Experimental *µ* (cm^−1^)					
PS	0.1822	0.1119	0.0862	0.0664	0.0614
Δ% (XCOM)	0.9174	1.0619	0.6678	1.7497	0.4408
PS-PbO-K					
PS-PbO-K 10	0.6298	0.1306	0.0914	0.0683	0.0637
Δ% (XCOM)	1.3453	0.7991	0.2480	0.6057	0.3769
PS-PbO-K 15	0.9257	0.1523	0.1005	0.0740	0.0688
Δ% (XCOM)	0.7904	0.7515	0.1486	0.9060	0.4843
PS-PbO-K 25	1.5127	0.1786	0.1086	0.0785	0.0726
Δ% (XCOM)	0.0132	0.7088	0.8672	0.8026	0.0517
PS-PbO-K 35	2.2598	0.2201	0.1257	0.0860	0.0798
Δ% (XCOM)	0.6357	0.9009	0.2173	0.8994	1.0797
PS-PbO-A					
PS-PbO-A 10	0.7337	0.1486	0.1021	0.0750	0.0689
δ%	16.4973	13.7825	11.7068	9.8097	8.1633
PS-PbO-A 15	1.0800	0.1693	0.1115	0.0810	0.0740
δ%	16.6685	11.1622	10.9453	9.4595	7.5581
PS-PbO-A 25	1.7940	0.2064	0.1240	0.0867	0.0792
δ%	18.5959	15.5655	14.1805	10.4459	9.0909
PS-PbO-A 35	2.7170	0.2563	0.1421	0.0963	0.0885
δ%	20.2319	16.4471	13.0469	11.9767	10.9023
PS-PbO-B					
PS-PbO-B 10	0.7554	0.1524	0.1043	0.0767	0.0706
δ%	19.9428	16.6922	14.1138	12.2987	10.832
PS-PbO-B 15	1.0982	0.1723	0.1132	0.0816	0.0749
δ%	18.6345	13.1320	12.6368	10.2703	8.8663
PS-PbO-B 25	1.9161	0.2217	0.1327	0.0919	0.0833
δ%	26.6675	24.1321	22.1915	17.0701	14.7383
PS-PbO-B 35	2.8207	0.2660	0.1493	0.1007	0.0928
δ%	24.8208	20.8542	18.7749	17.0930	16.2907

As anticipated, *μ* decreases as γ-ray energy increases. Table 4 demonstrates a sharp decrease in the LAC when energy increases from 0.059 to 0.662 MeV and a slow decrease for the remaining energies^35^. In the low-energy range, photons primarily interact with matter through the photoelectric effect. The photoelectric coefficient depends on Z^4^/E^3^, which explains the sharp decrease of µ of the PS-PbO composite with increasing energy in the low-energy range. As a result of the energy dependence of the photoelectric coefficient, it decreases by increasing photon energy^36–38^. In this study, the low-energy spectrum lacks photon energies around 88 keV, corresponding to the K-edge of lead (Pb). Consequently, the graph fails to capture the sharp increase in the linear attenuation coefficient (LAC) between 80 and 100 keV, where photoelectric absorption dominates at energies just beyond the binding energy of k-shell electrons^39^. At higher energies, despite Compton scattering and pair-production interactions prevailing, the LAC decreases with increasing incident photon energy. For all energy ranges, the cross-section of the different interaction types is directly proportional to the atomic number Z, which explains the increase of the LAC with increasing the PbO concentration in the composite.

At energy level 0.059 MeV, the Linear Attenuation Coefficient (LAC) of the PS-PbO composite increases proportionally with the filler weight percentage, reflecting the direct proportionality to Z^4^ (Photoelectric). Specifically, the LAC values increased by factors of 1.5, 2.5, and 3.5 as the filler weight percentage increased from 10 to 15%, 25%, and 35%, respectively. At higher energy levels, the LAC continued to increase with higher PbO weight percentages; however, the increase was not proportional to the filler factor as it follows a Z proportionality. The relative increase (δ%) in LAC due to particle size variations revealed that for a given PbO weight percentage and the same incident gamma-ray energy, there was a 20% and 25% increase (δ%) for PS-PbO-A and PS-PbO-B compared to PS-PbO-K at high concentration and low energy. In the high-energy range, the relative increase (δ%) for PS-PbO-A and PS-PbO-B over PS-PbO-K was approximately 10% and 16%, respectively.

**Figure 4:**
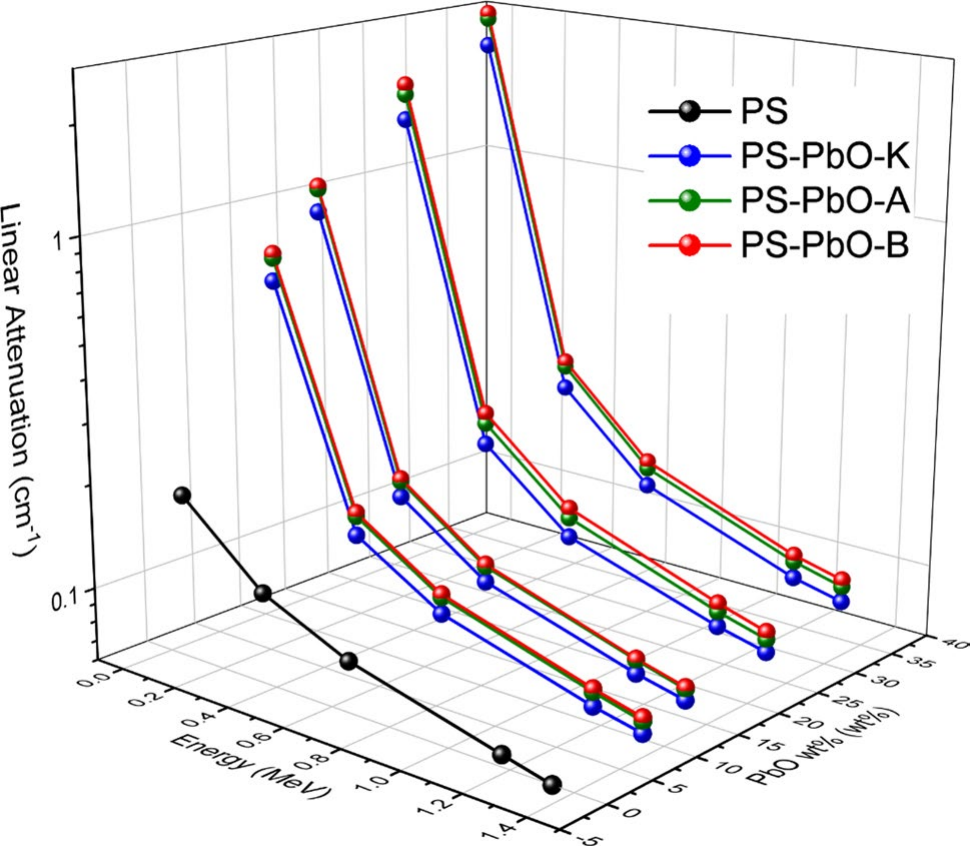
Experimental linear attenuation coefficient values *μ* of PS-PbO composites as a function of Gamma-ray energy and PbO weight fraction.

Figure 4 illustrates the LAC of composites with varying PbO sizes across different photon energies. The slopes in Fig. 4 show a sharp decrease in LAC for energies ranging from 0.059 to 0.662 MeV, followed by a more gradual decrease for energies between 0.6 and 1.34 MeV. The aforementioned relationship with photon energy, PbO mass fraction, and the influence of PbO size is quite obvious. Through other meanings, the investigated composite absorbs photons more efficiently as the filler particle size decreases, especially at low energies. Particles’ number per unit weight and density increase, when particle size decreases, and nanoparticle spacing diminishes. As a consequence of the high ratio of surface to volume, the particles were spread evenly on a larger surface area of the polymer matrix and hence increasing the probability of interaction of the incident photons^40^. Many researchers have previously discussed the relationship between the particle size of filler and the radiation attenuation ability of composite. In particular, a comparison of micro and nanoparticles reported that nano-sized particles possessed higher dispersion forces and achieved higher responses in radiation shielding^41–44^. The current investigation validated this finding.

### Total molecular cross section and effective atomic cross section

Using Eqs. (7) and (8), total molecular cross-section *σ*_*mol*_ and total atomic cross-section *σ*_*atm*_ were calculated. According to the XCOM database, the theoretical values of *μ* were estimated. Experimental values of *σ*_*mol*_ and *σ*_*atm*_ for PS and PS-PbO composite, in addition to theoretical ones, are listed in Table 5. The results indicate that the measured total molecular cross-section *σ*_*mol*_ and total atomic cross-section *σ*_*atm*_ of PS-PbO-K are in fair accordance with the theoretical values. For the energy range used in this study, both cross-sections were analyzed. Table 5 reveals a similar trend of the LAC for *σ*_*mol*_ and *σ*_*atm*_, which decrease with increasing photon energy, presenting a sharp decrease in the low energy range and a slow decrease in the high energy range. With increasing photon energy, photoelectric absorption is reduced, while Compton scattering and pair production predominate. Table 5 demonstrates that the molecular *σ*_*mol*_ and atomic *σ*_*atm*_ cross-sections of PS-PbO composites increase with a greater weight percent of PbO in the composite. This is because an increase in the weight percent of PbO leads to an increase in the number of lead atoms and electrons within the composite, which subsequently enhances the probability of γ-ray interaction^45^. Furthermore, results in Table 5 reveal that a reduction in PbO size increases *σ*_*mol*_ and *σ*_*atm*_, and PS-PbO composites with PbO-B consistently exhibit greater values of *σ*_*mol*_ and *σ*_*atm*_ compared to other PbO particles, even when the PbO weight percent and the photon energy are held constant^46^.

**Table 5:** Cross-section values for total molecular $\sigma_{mol}$ and atomic cross-sections $\sigma_{atm}$ of PS and PS-PbO composites at varying PbO concentrations and different photon energies.

Sample	Energy (keV)	Total molecular cross-section σ_mol_ × 10^−24^ (cm^2^/molecule)				Effective atomic cross-section σ_atm_ × 10^−24^ (cm^2^/atom)			
PbO (wt%)		PS (Experimental)			PS (XCOM)	PS (Experimental)			PS (XCOM)
0 wt%	59.53	30.8194			30.816	1.9262			1.926
	356.01	18.5574			18.5494	1.1598			1.1593
	661.66	14.3513			14.3504	0.897			0.8969
	1173.23	10.9373			10.9367	0.6836			0.6835
	1332.5	10.2455			10.2457	0.6403			0.6404

PbO (wt%)	Energy (keV)	PS-PbO-K	PS-PbO-A	PS-PbO-B	XCOM	PS-PbO-K	PS-PbO-A	PS-PbO–B	XCOM
10 wt%	59.53	106.49	119.28	121.60	107.93	11.32	12.68	12.92	11.47
	356.01	22.08	24.16	24.53	22.26	2.35	2.57	2.61	2.37
	661.66	15.45	16.60	16.79	15.49	1.64	1.76	1.78	1.65
	1173.23	11.55	12.19	12.35	11.48	1.23	1.30	1.31	1.22
	1332.5	10.77	11.20	11.37	10.73	1.14	1.19	1.21	1.14
15 wt%	59.53	148.67	169.30	171.89	149.85	19.05	21.70	22.03	19.20
	356.01	24.46	26.54	26.97	24.28	3.13	3.40	3.46	3.11
	661.66	16.14	17.48	17.72	16.12	2.07	2.24	2.27	2.07
	1173.23	11.88	12.70	12.77	11.78	1.52	1.63	1.64	1.51
	1332.5	11.05	11.60	11.72	11.00	1.42	1.49	1.50	1.41
25 wt%	59.53	241.44	279.86	283.65	241.44	41.50	48.11	48.76	41.50
	356.01	28.50	32.20	32.82	28.70	4.90	5.53	5.64	4.93
	661.66	17.33	19.34	19.64	17.48	2.98	3.33	3.38	3.01
	1173.23	12.53	13.52	13.60	12.43	2.15	2.33	2.34	2.14
	1332.5	11.59	12.35	12.33	11.58	1.99	2.12	2.12	1.99
35 wt%	59.53	342.82	405.40	408.05	345.04	73.93	87.42	87.99	74.41
	356.01	33.39	38.24	38.48	33.69	7.20	8.25	8.30	7.27
	661.66	19.07	21.20	21.60	19.02	4.11	4.57	4.66	4.10
	1173.23	13.05	14.37	14.57	13.16	2.81	3.10	3.14	2.84
	1332.50	12.11	13.21	13.42	12.24	2.61	2.85	2.90	2.64

Equations (9), (10), and (11) were used to compute the total electronic cross-section (*σ*_*el*_), effective atomic number (*Z*_*eff*_), and the effective electron density (*N*_*eff*_) of the PS-PbO composites. Additionally, the WinX-Com program was used to compute the mass attenuation coefficients of each element in the samples^47^. The shielding parameters (*σ*_*el*_, *Z*_*eff*_, and *N*_*eff*_) were then estimated and presented in Table 6. The results show that raising the PbO concentration or reducing the PbO particle size in composites leads to an increase in their values. Moreover, the values of *σ*_*el*_, *Z*_*eff*_, and *N*_*eff*_ decrease with an increase in photon energy, indicating that composite materials with significant *Z*_*eff*_ effectively capture incident γ-ray photons. Furthermore, *N*_*eff*_ gradually increases as the Z_eff_ of the composites increases^48^. *Z*_*eff*_ is an essential parameter that reflects various features of a substance, such as its chemical composition and shielding properties in radiation therapy applications. A greater electron density *N*_*eff*_ increases the probability of energy transfer between the photon and the electron, and deposition of energy in the composite^49^.

**Table 6:** Total electronic cross-section $\sigma_{el}$, effective atomic number $Z_{eff}$ and electron density $N_{eff}$ of PS and PS-PbO at different PbO concentrations and various photon energies.

Sample	Energy (keV)	σ_el_ (cm^2^/electron)	Z_eff_	N_eff_ × 10^23^ (electrons/g)
		Theoretical		
PS	59.53	0.5482	3.5136	3.2506
	356.01	0.3314	3.4985	3.2366
	661.66	0.2563	3.5000	3.2379
	1173.23	0.1953	3.4997	3.2377
	1332.5	0.1830	3.5001	3.2380

PbO wt%	Energy (keV)	PS-PbO-K		
10	59.53	1.0137	11.3159	5.8308
	356.01	0.3507	6.7463	3.4762
	661.66	0.2606	6.3169	3.2550
	1173.23	0.1967	6.2037	3.1966
	1332.5	0.1841	6.1957	3.1925
15	59.53	1.2667	15.1605	6.2959
	356.01	0.3611	8.6161	3.5781
	661.66	0.2630	7.8523	3.2609
	1173.23	0.1974	7.6462	3.1753
	1332.50	0.1847	7.6311	3.1691
25	59.53	1.8195	22.8104	6.6524
	356.01	0.3840	12.8451	3.7462
	661.66	0.2682	11.2026	3.2671
	1173.23	0.1990	10.7363	3.1311
	1332.5	0.18600	10.7010	3.1208
35	59.53	2.4448	30.4349	6.6397
	356.01	0.4099	17.7241	3.8667
	661.66	0.2741	14.9657	3.2649
	1173.23	0.2008	14.1382	3.0844
	1332.5	0.1875	14.0741	3.0704

### Half and tenth value layers and heaviness

In terms of shielding, the half-value layer HVL and the tenth-value layer TVL are important parameters described as the thicknesses or layers of an absorber or a shield that reduce the radiation intensity by half and a tenth of the main intensity, respectively. According to Eqs. (12) and (13), HVL and TVL are inversely proportional to the mass attenuation coefficient.

12 $$HVL = \frac{Ln2}{\mu }$$

13 $$TVL = \frac{Ln10}{\mu }$$

The experimental values of HVL and TVL for all PS-PbO composites at all energies are listed in Table 7. These parameters increase as photon energy increases from 0.06 to 1.33 MeV. According to the results obtained, increasing the PbO content will indeed expect the development of a more compact shield^50,51^.

**Table 7:** The Half and Tenth value layers of PS-PbO-K, PS-PbO-A, and PS-PbO-B.

Energy (MeV)	0.059	0.356	0.661	1.173	1.332	0.059	0.356	0.661	1.173	1.332
	Half Value Layer, HVL (cm)					Tenth Value Layer ,TVL (cm)				
PS	3.80	6.19	8.04	10.44	11.29	12.64	20.58	26.71	34.68	37.50
Pb	0.012	0.213	0.554	0.989	1.088	0.039	0.706	1.839	3.286	3.613
PS-PbO-K 10	1.10	5.31	7.58	10.15	10.88	3.66	17.63	25.19	33.71	36.15
PS-PbO-K 15	0.75	4.55	6.90	9.37	10.07	2.49	15.12	22.91	31.12	33.47
PS-PbO-K 25	0.46	3.88	6.38	8.83	9.55	1.52	12.89	21.20	29.33	31.72
PS-PbO-K 35	0.31	3.15	5.51	8.06	8.69	1.02	10.46	18.32	26.77	28.85
PS-PbO-A 10	0.94	4.66	6.79	9.24	10.06	3.14	15.50	22.55	30.70	33.42
PS-PbO-A 15	0.64	4.09	6.22	8.56	9.37	2.13	13.60	20.65	28.43	31.12
PS-PbO-A 25	0.39	3.36	5.59	7.99	8.75	1.28	11.16	18.57	26.56	29.07
PS-PbO-A 35	0.26	2.70	4.88	7.20	7.83	0.85	8.98	16.20	23.91	26.02
PS-PbO-B 10	0.92	4.55	6.65	9.04	9.82	3.05	15.11	22.08	30.02	32.61
PS-PbO-B 15	0.63	4.02	6.12	8.49	9.25	2.10	13.36	20.34	28.22	30.74
PS-PbO-B 25	0.36	3.13	5.22	7.54	8.32	1.20	10.39	17.35	25.06	27.64
PS-PbO-B 35	0.25	2.61	4.64	6.88	7.47	0.82	8.66	15.42	22.87	24.81

For the low-energy beam 0.06 and 0.356 MeV, the HVL of PS-PbO-K 35 wt% are 0.31 and 3.15 cm, respectively, while for PS-PbO-B 35 wt% are 0.25 and 2.61 cm. This represents a relative decrease in HVL of 19% and 17%, respectively. In the high-energy range, the relative decrease in HVL is approximately 15%. This decrease is attributed solely to the effect of nanoparticle size. The observed nano-size effect of PbO particles indicates that the nano-size filler with the smallest dimensions provides exceptional shielding performance.

The attractiveness of polymer composites stems from their light weight. In the application of any shielding material, heaviness is also a crucial consideration. The percent heaviness of polystyrene reinforced by high-density lead oxide, as well as some conventional shielding materials, as calculated by the following Eq. (14):

14 $$Heaviness = \frac{Density\;of\;composite}{{ Density\;of\;Lead}} \times 100 \left( \% \right)$$

As illustrated in the chart, the relative densities of concrete, barite, and steel are 20.28%, 39.50%, and 69.22% of lead, respectively, with lead serving as the reference at 100%. For PS-PbO composites, PS-PbO-B at 35 wt% has a density equivalent to 12.7% of lead, while polystyrene has a density equivalent to 9.1% of lead. Compared to traditional shielding materials such as lead, barite, and steel, polymer nanocomposites provide a lighter alternative for shielding applications^52^.

A mass comparison was conducted between one Half-Value Layer (HVL) of lead and PS-PbO-B 35 wt%, both with the same surface area (*S*), at an energy of 1.332 MeV:

$$\frac{{D_{composite} }}{{D_{Lead} }} = 0.127 = \frac{1}{8}; \frac{{HVL_{Lead} }}{{HVL_{composite} }} = { }\frac{1}{7};$$

$$\frac{{D_{composite} }}{{D_{Lead} }} = { }\frac{{\frac{{m_{composite} }}{{V_{composite} }}}}{{\frac{{m_{Lead} }}{{V_{Lead} }}}} = \frac{{\frac{{m_{composite} }}{{S \times HVL_{composite} }}}}{{\frac{{m_{Lead} }}{{S \times HVL_{Lead} }}}} = \frac{{m_{composite} }}{{m_{Lead} }} \times \frac{{HVL_{Lead} }}{{HVL_{composite} }} = \frac{{m_{composite} }}{{m_{Lead} }} \times \frac{1}{7} = \frac{1}{8}$$

$$\to \frac{{m_{composite} }}{{m_{Lead} }} = { }\frac{7}{8} = 0.85.$$

$$m_{PbO} = 35\% \times m_{composite} = 0.35 \times 0.85 \times m_{Lead} = 0.3 \times m_{Lead}$$

This result indicates a 70% reduction in the mass of Lead when choosing an *HVL* of the polymer nanocomposite.

## Conclusion

This research investigated the impact of particles’ size and their concentration on the PS-PbO gamma rays shielding ability. LAC, HVL, TVL, total molecular and atomic cross-sections (*σ*_*mol*_, *σ*_*atm*_), effective atomic numbers *Z*_*eff*_, as well as effective electron densities *N*_*eff*_ of the composite, were all evaluated. The experimental values revealed that the gamma-ray shielding performance of PS-PbO composites was improved in both low and high energy ranges by raising PbO concentration, which is in accordance with the theory in increasing the percentage of Pb increases the number of electrons within the composite, which subsequently enhances the probability of γ-ray interaction. The decline in photoelectric interaction and the prevalence of the Compton effect with increasing incident photon energy explain the decrease of *σ*_*mol*_ and *σ*_*atm*_. The influence of PbO size on *µ* led to a 25% improvement of the attenuation capability in the low-energy range and a 16% improvement in the high-energy range for PS-PbO-B over PS-PbO-K. Moreover, decreasing the size of the particle increased *σ*_*e*_, *Z*_*eff*_, and *N*_*eff*_, a consequence of the high proportion of surface to volume and the occupancy of outer electrons over a wide area, which increase the interaction probability with Gamma photons. Furthermore, the study demonstrated that HVL becomes thinner as particle size decreases. Specifically, the HVL of PS-PbO-B 35 wt% contains 70% less lead mass compared to a typical lead shield and is 12.75% lighter. These findings indicate that the new PS-PbO nanocomposite, consisting of PbO nanoparticles produced by a high-speed ball mill and incorporated into polystyrene using a roller mixer and pressing, is a cost-effective and non-toxic material. This composite serves as an efficient, lightweight shield with a substantial attenuation effect on gamma radiation.

## Data Availability

Access to the data presented in this paper can be provided and for this and any further inquiries about our work please contact the corresponding authors.
